# The Book World of Medicine and Science

**Published:** 1906-10-27

**Authors:** 


### THE BOOK WORLD OF MEDICINE AND SCIENCE.

Indications for Operation in Disease of the Internal Organs. By Professor Hermann Schlesinger. Authorised English translation by Keith W. Monsarrat. (Bristol: John Wright and Co. 1906. Pp. xv. and 498. Price 9s. 6d. net.)

The plan of the book is to give a short account of the etiology of the disease under consideration, followed by a short account of the pathological anatomy and of the clinical course. The diagnosis and differential diagnosis are next discussed and the indications for and against operation. The prognosis is given under the headings of risks and results of operation on the one hand, and the forecast if the patient be left without operation on the other hand. There are twenty-five chapters treating in this manner of the various parts of the body accessible to surgical interference, and there are three appendices which deal respectively with the indications for the induction of premature labour, operations on diabetics, and the general influence of operations on the body, this last article being written by Dr. Julius Schnitzler. Professor Schlesinger is well known as the Extraordinary Professor of Medicine in the University of Vienna, and the work is therefore an authoritative and adequate exposition of Austrian medicine towards modern surgical procedure. It shows that the Austrian physicians fully appreciate the value of surgery, whilst they maintain a healthy attitude of restraint towards merely experimental operations in the same manner as do their colleagues in England. The information contained is for the most part accurate, well arranged, and up to date. The least satisfactory sections perhaps are those which deal with duodenal ulcer and perforating gastric ulcer. Professor Schlesinger says frankly that perforation of the stomach is rare in Vienna, whilst in the case of duodenal perforation he lays too much stress upon symptoms and too little upon the signs which are all important in arriving at the correct diagnosis needed for immediate and successful operation. In intussusception, too, he teaches that the subacute and chronic forms are not very uncommon and thus gives the practitioner an excuse for waiting. He should lay emphasis on the rarity of these forms and the necessity for instant operation when the diagnosis is established. Mr. Keith Monsarrat has performed his work of translation excellently. The text is free from German idioms and is rendered into fluent English. The title alone is a little misleading, for the book contains chapters on the diseases of the spinal column, of the joints, of the bones, and of the peripheral nerves. None of these structures are internal organs in the English sense of the expression, and the more accurate rendering, therefore, would perhaps be indications for operations in disease coming under the care of the physician or into the medical wards. A slender bibliography of not very recent work, but with the references verified, is appended to each chapter. There is a good index.

Clinical Lectures on Neurasthenia. By Thos. D. Savill, M.D. Third edition. (London: Henry J. Glaisher, 1906. Pp. xvi. and 216. Price 7s. 6d. net.)

Dr. Savill's lectures are eminently readable and interesting. Considering the importance of the subject with which they deal, and the manner of the author's treatment of it, there can be no surprise at the fact that his book has reached a third edition. The clinical basis on which the conclusions are based is a very considerable one and has been subjected to careful and critical analysis. Hence the reader is in a position to appreciate the author's point of view, even though he may not at all points assent to the arguments advanced. It is of especial interest to note the emphasis which is placed on the wide diffusion of neurasthenia and the contradiction of the error that the disease is restricted to the more educated members of the community. Some may be expected to urge that Dr. Savill throws rather a wide net and that some of his clinical records are hardly examples of neurasthenia in the ordinary meaning of that term. Similarly it may be questioned whether he is entirely successful in the distinctions he endeavours to establish between neurasthenia and hysteria. But whatever controversies might be instituted on these points, there can be no difference of opinion on the practical interest and value of the lectures, and the discussion on the subject of treatment merits general appreciation. Completeness is lent to the volume by an appendix dealing with the historical aspect of the subject and by a detailed bibliography; both of these have been prepared by Dr. Agnes Savill, who is to be complimented on the success and thoroughness of her work.

Clinical Studies in the Treatment of the Nutritional Disorders of Infancy. By Ralph Vincent, M.D., M.R.C.P. (London: Baillière, Tindall and Cox, 1906. Pp. viii. and 84.

In these "studies" are presented the details of twenty cases of ill-nourished infants and the milk prescriptions adopted in each instance to meet the occasion. A diary devoted to a record of an infant's weight and the colour of the stools is hardly entertaining reading, and when repeated page after page and extended to twenty successive cases, becomes almost unendurable. Nevertheless, to those who have patience there may be found in this small volume a number of useful hints on the important subject of infant feeding.

Some Physiological Actions of Ergot. By H. H. Dale, M.A., B.C. (Reprinted from the *Journal of Physiology.*)

This is an elaborate pharmacological study based upon experiments conducted in the Wellcome Research Laboratories. The full appreciation of it is doubtless only possible to those whose training in modern physiological methods has been considerable. But all may recognise the industry and ingenuity which characterise the work, and may be sure that practical medicine will, sooner or later, be the richer for the vigour and perseverance with which such studies as the present are nowadays being conducted.

